# The clinical significance of T cell infiltration and immune checkpoint expression in central nervous system germ cell tumors

**DOI:** 10.3389/fimmu.2025.1536722

**Published:** 2025-01-31

**Authors:** Jiajun Zhou, Wenhao An, Lei Guan, Jinyu Shi, Qiaozhen Qin, Shuai Zhong, Zheng Huang, Rui Liu, Chenxing Wu, Zhong Ma, Xueling Qi, Xiaoxia Jiang, Yan Wang, Shouwei Li

**Affiliations:** ^1^ Department of Neurosurgery, Sanbo Brain Hospital, Capital Medical University, Beijing, China; ^2^ Department of Neuroimmunology, Beijing Institute of Basic Medical Sciences, Beijing, China; ^3^ Department of Pathology, Sanbo Brain Hospital, Capital Medical University, Beijing, China

**Keywords:** germ cell tumor, tumor microenvironment, tumor-infiltrating lymphocyte (TILs), immune checkpoint, Foxp3, CTLA-4, PD-1, PD-L1

## Abstract

**Background:**

Primary central nervous system germ cell tumors (CNS GCTs) are rare intracranial malignancies, and their tumor microenvironment plays a crucial role in tumor initiation and progression. However, the specific characteristics of the immune microenvironment and their clinical significance remain poorly understood.

**Methods:**

This study included 93 paraffin-embedded tissue samples from 90 patients diagnosed with CNS GCTs. Immunohistochemistry and immunofluorescence staining were used to assess the infiltration patterns of T cell subsets (CD3+, CD4+, CD8+, Foxp3+) and the expression levels of immune checkpoints (CTLA-4, PD-1, PD-L1). Additionally, the study explored the relationship between these immune features and the patient’s clinical characteristics and prognosis.

**Results:**

The study revealed that germinomas exhibited significantly higher infiltration of CD4+ and Foxp3+ T cells compared to non-germinomatous GCTs (NGGCTs). Additionally, CTLA-4 expression was detected in 58.06% of cases, while PD-1 and PD-L1 were expressed in over 90%, with higher CTLA-4 levels in germinomas and elevated PD-L1 levels in NGGCTs. T cell infiltration was positively correlated with immune checkpoint expression, particularly in germinomas. The results also highlighted the strong immunosuppressive nature of the CNS GCTs’ tumor microenvironment. Furthermore, T cell infiltration and immune checkpoint expression were closely associated with clinical characteristics and prognosis. Notably, PD-1 expression was identified as an independent prognostic factor for progression-free survival (PFS) and recurrence-free survival (RFS).

**Conclusion:**

Our study highlighted the distinct characteristics of T cell infiltration and the significant expression of immune checkpoints in CNS GCTs, revealing the highly heterogeneous and immunosuppressive nature of the tumor microenvironment. PD-1 expression was identified as an independent prognostic predictor, offering a foundation for enhancing risk stratification in CNS GCT patients. These findings also support the potential for future clinical applications of immune checkpoint inhibitors, such as PD-1 monoclonal antibodies.

## 1 Introduction

Germ cell tumors can arise not only in the gonads but also in other midline regions of the body, including the central nervous system. Primary central nervous system germ cell tumors (CNS GCTs) are a rare form of intracranial malignancy, typically classified into two groups based on histopathological characteristics: germinomas and non-germinomatous germ cell tumors (NGGCTs). Germinomas comprise approximately 64-75% of CNS GCTs, while more than half of NGGCTs are mixed germ cell tumors containing various histological components (1). Germinomas are highly responsive to both radiotherapy and chemotherapy, and current treatment strategies rely on a combination of these modalities. However, significant clinical challenges persist. These tumors often occur in young patients, and the adverse effects of radiotherapy and chemotherapy, such as hormonal imbalances and long-term neurological and cognitive dysfunction, are a serious concern (2–4). Furthermore, some patients exhibit treatment resistance and disease recurrence, leading to poor disease control and limited improvements in prognosis following recurrence (5). This underscores the urgent need for new risk stratification methods and innovative therapeutic approaches to improve patient outcomes.

In recent years, the tumor microenvironment has gained significant attention in cancer research due to its pivotal role in tumorigenesis and malignant progression (6). Numerous studies have documented the presence of abundant immune cell infiltration and immune checkpoint expression in gonadal germ cell tumors (7–12). Similarly, research on CNS GCTs has identified comparable patterns of immune infiltration (13–17). Immune checkpoint molecules, such as the PD-1/PD-L1 axis and CTLA-4, are crucial in modulating T cell responses to tumor antigens (18, 19). The interaction between these molecules and their ligands can suppress anti-tumor immune responses, facilitating tumor immune evasion (18–20). As a result, immune checkpoint inhibition therapy has become a promising strategy for treating malignant tumors. However, research specifically focused on CNS GCTs remains limited, and the relationship between immune cell infiltration within the tumor microenvironment and tumor development, progression, and patient prognosis is not yet fully understood. Moreover, data on CTLA-4 expression and its influence on the prognosis of CNS GCT patients are sparse. Further investigation and clinical studies are needed to evaluate the potential of immune checkpoint inhibition therapy for CNS GCTs.

To address these gaps, we conducted a comprehensive analysis of T cell infiltration characteristics and immune checkpoint expression in our CNS GCTs cohort. We explored the correlation between these factors and their association with clinical characteristics and patient prognosis. Our findings unveiled the distinct tumor microenvironment of CNS GCTs, providing insights into the potential application of immune checkpoint blockade (ICB) therapy for this disease.

## 2 Methods

### 2.1 Patients and tumor specimens

This study included a cohort of CNS GCT patients consecutively diagnosed pathologically at Sanbo Brain Hospital, Capital Medical University, Beijing, China, between January 2020 and January 2022. We excluded patients who met any of the following criteria: patients diagnosed with non-central nervous system germ cell tumors; patients with a history of other malignant diseases; patients with incomplete clinical data. Besides, two patients whose tissue samples were obtained through biopsy were excluded, as the samples were too small and had insufficient tumor components for analysis. A total of 90 patients were ultimately included: 62 Germinoma cases (60 primary cases and 2 recurrent cases) and 28 NGGCT cases (26 primary cases and 2 recurrent cases). Three primary NGGCT patients experienced disease recurrence and underwent secondary surgery at our center, allowing us to collect both primary and recurrent tissue samples. As a result, the study incorporated 93 paraffin-embedded tissue samples. Of the specimens, 54 were obtained through surgical resection, while 39 were acquired via biopsy. All diagnoses were histologically confirmed and reviewed by two or more pathologists, including Prof. Xueling Qi, according to World Health Organization (WHO) classification criteria. Imaging data and clinical information—such as general demographics, medical history, physical examination findings, treatment course, and tumor marker levels—were retrieved from the hospital’s electronic medical record system. Central nervous system dissemination was defined as cases with intracranial and/or spinal dissemination. Diagnostic thresholds for tumor markers were set at 7 IU/L for β-HCG (human chorionic gonadotropin) and 5 ng/ml for AFP (alpha-fetal protein), based on clinical testing standards. Demographic and histological characteristics of all patients are detailed in **Supplementary Table S1** and **Supplementary Figure S1**. Histopathological diagnoses of the 31 samples from 28 NGGCT patients are summarized in **Supplementary Table S2**.

Survival and prognostic information were obtained through in-hospital records and telephone follow-ups, which continued until February 2024. Five patients (three with Germinoma and two with NGGCT) were lost to follow-up, resulting in a loss rate of 5.6%. Of the 90 patients, 83 (92.22%) underwent combined chemotherapy and radiotherapy after histological diagnosis from the initial surgery. Prognostic analyses for Germinoma and NGGCT patients are shown in **Supplementary Figure S1**. This study adhered to the Declaration of Helsinki and received approval from the Ethics Committee of Sanbo Brain Hospital, Capital Medical University. Written informed consent for specimen storage was obtained from all patients or their legal representatives.

### 2.2 Immunohistochemical staining

The antibodies used in this study, along with vendors, clone numbers, and dilution ratios, are listed in **Supplementary Table S3**. Tissue samples were fixed, dehydrated, and sectioned at a thickness of 5 μm. Following dewaxing, the sections were rehydrated in a series of graded ethanol solutions. For immunohistochemistry staining, antigen retrieval was performed using high-pressure thermal repair in citrate buffer (pH 6.0, ZLI-9064, ZSGB-BIO) and Tris/EDTA buffer (pH 9.0, ZLI-9068, ZSGB-BIO) for 2.5 minutes, following the recommendations of the primary antibody manufacturer. Endogenous peroxidase activity was blocked using 3% hydrogen peroxide. The sections were then incubated overnight at 4°C with the appropriate monoclonal primary antibody, followed by incubation with the secondary antibody (PV-9000, ZSGB-BIO). Slides were developed using diaminobenzidine substrate solution (ZLI-9018, ZSGB-BIO) and counterstained with hematoxylin (G1120, Solarbio). Tonsil tissue samples served as positive controls, as recommended by the antibody manufacturer, while PBS was used as a negative control in place of the primary antibody.

For immunofluorescence staining, slides were similarly dewaxed, antigen retrieval was performed, and endogenous peroxidase activity was removed using the same protocols. The sections were blocked for 1 hour in a solution containing 10% normal goat serum and 0.3% Triton X-100 (9002-93-1, Solarbio). The slides were then incubated overnight at 4°C with the primary antibodies. After washing with PBS, the sections were incubated with the appropriate secondary antibodies conjugated with Neon520 and Neon570 (DFT52100; DFT57100, HISTOV) at 37°C for 10 minutes, followed by a 1-minute incubation with Neon520 and Neon570. Finally, the slides were mounted using a DAPI-containing mounting medium (ab104139, Abcam) and observed under a microscope after being stored in the dark. Tonsil tissue samples served as positive controls, while PBS replaced the primary antibody for the negative control.

### 2.3 Quantification of T cell subset infiltration and immune checkpoint expression levels

Slides were photographed and digitized using the ZEISS Imager Z2 microscope, and the images were analyzed with Image J (Version 1.54d). For NGGCTs, tumor areas were microscopically divided based on marker-specific staining for different histological types. Infiltrating immune cells were analyzed by selecting five representative tumor-rich areas per sample, viewed under high-power fields (HPF, 40X). The proportion of positively stained cells relative to the total nuclear cell area was calculated semi-quantitatively, with accuracy confirmed through screenshot evaluations. Similarly, for immune checkpoint expression, five representative areas per sample were selected and analyzed under HPF (40X), using the same method. The percentage of positively stained cells relative to the total nuclear cell area was used to calculate the proportional score (PS), with the estimated proportions categorized into five subgroups: (1) 1% - 5%, (2) 6% - 10%, (3) 11% - 25%, (4) 26% - 50%, and (5) greater than 50%.

Staining intensity was assessed using the IHC Profiler plugin in Image J, with grayscale values ranging from 0-60 for strong, 61-120 for moderate, 121-180 for weak, and 181-236 for unstained areas. The intensity score (IS) was determined by comparing staining intensity to control cells and categorized as follows: 0 (absent), 1 (weak), 2 (moderate), and 3 (strong). The total immunostaining score (TIS) was calculated by multiplying the PS by the IS to assess the expression of PD-1, PD-L1, and CTLA-4. Screenshot evaluations were also used to confirm the accuracy of these calculations.

In NGGCT cases, for immune cell infiltration analysis, the histological component with the highest CD3+ cell infiltration was selected to represent the tumor for subsequent comparisons. Similarly, for immune checkpoint expression evaluation, the tumor’s histological component with the highest TIS was chosen for further comparison. Membranous staining in 1% or more of tumor cells was considered positive for PD-1, PD-L1, and CTLA-4 expression.

### 2.4 Statistical analysis

Statistical analyses were conducted using R software (Version 4.3.0) and IBM SPSS Statistics version 26. Associations between categorical variables were assessed using the chi-square test or Fisher’s exact test. Continuous variable distributions were analyzed with the non-parametric Mann-Whitney U test and Kruskal-Wallis test as appropriate. Differences in immune cell infiltration abundance among groups were evaluated using the Wilcoxon test, while correlations between the two groups were examined using Pearson correlation analysis. The correlation between immune cell infiltration abundance and immune checkpoint expression scores was analyzed with Spearman correlation analysis. Survival analysis utilized Kaplan-Meier estimates and the log-rank test. ROC curves were used to determine optimal cut-off values for categorizing high and low infiltration groups in the correlation analysis between immune cell infiltration abundance and prognosis. Variables with a p-value < 0.05 in univariate analysis were included in the Cox proportional hazards regression model. A p-value < 0.05 was considered statistically significant.

## 3 Results

### 3.1 Infiltration characteristics of T cell subsets in CNS GCTs

**Figure 1:**
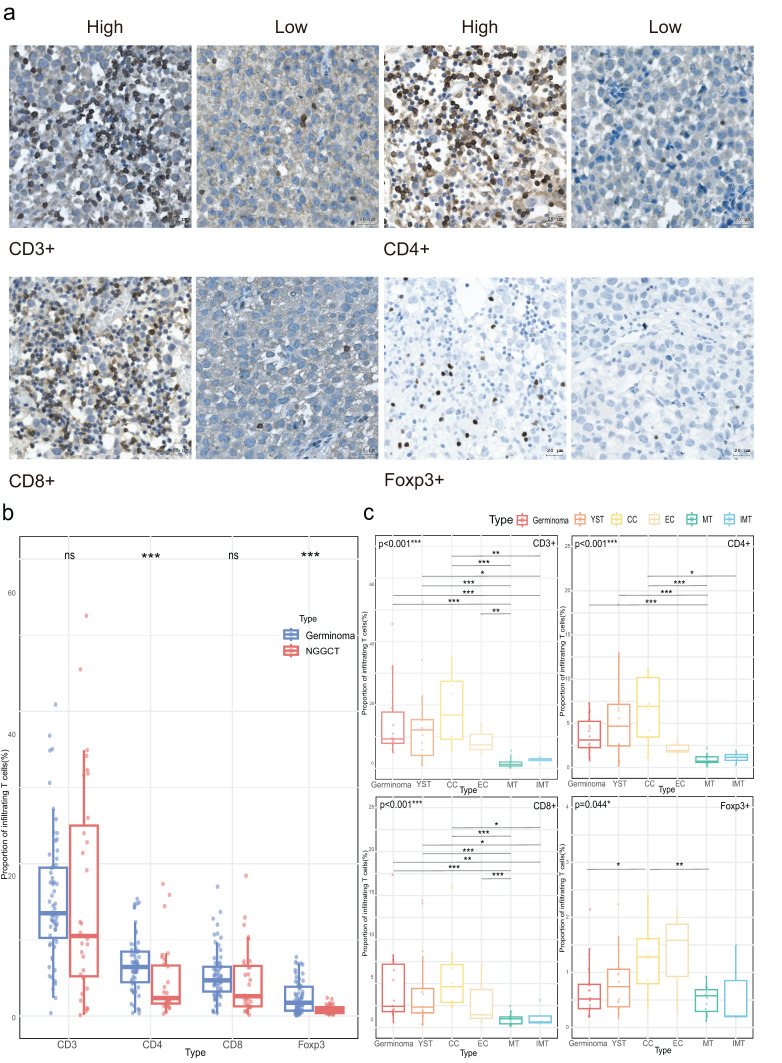
Density of tumor-infiltrating T cells in CNS GCTs. **(A)** Representative immunohistochemical images of T cell subset staining in CNS GCT specimens. Nuclei counterstained with hematoxylin (blue). Original magnification, x40. Scale bar: 20 μm. **(B)** Box plots of T cell subset infiltration in Germinomas and NGGCTs. **(C)** Box plots of T cell subset infiltration across histological subtypes in NGGCTs. *p<0.05, **p<0.01, ***p<0.001. ns, non- significant; YST, yolk sac tumor; CC, choriocarcinoma; EC, embryonal carcinoma; MT, mature teratoma; IMT, immature teratoma.

First, we quantified the infiltration abundance of CD3+, CD4+, CD8+, and Foxp3+ cells, with representative IHC staining images shown in **Figure 1A**. Across the CNS GCTs cohort, the median infiltration rates were: CD3+ (13.13%, range: 0.14%-52.56%), CD4+ (5.84%, range: 0.32%-17.36%), CD8+ (4.57%, range: 0.17%-18.42%), and Foxp3+ (1.19%, range: 0.06%-7.76%), with no significant difference between CD4+ and CD8+ levels (p=0.113). In germinomas, CD3+, CD4+, CD8+, and Foxp3+ cells infiltrated at 13.49% (range: 0.40%-40.91%), 6.44% (range: 0.37%-15.39%), 4.69% (range: 0.37%-16.99%), and 1.75% (range: 0.06%-7.76%), respectively, with CD4+ T cell levels being significantly higher than CD8+ cells (p=0.010, data not shown). In NGGCTs, the proportions were 10.51% (range: 0.14%-52.56%) for CD3+, 2.36% (range: 0.32%-17.36%) for CD4+, 2.60% (median, range: 0.17%-18.42%) for CD8+, and 0.74% (median, range: 0.20%-2.40%) for Foxp3+ with CD4+ and Foxp3+ significantly higher in germinomas than NGGCTs (p<0.001, **Figure 1B**). Among the histological components of NGGCTs, mature and immature teratomas demonstrated notably lower infiltration levels of T cells compared to other tumor components (**Figure 1C**).

### 3.2 Expression patterns of immune checkpoints in CNS GCTs

**Figure 2:**
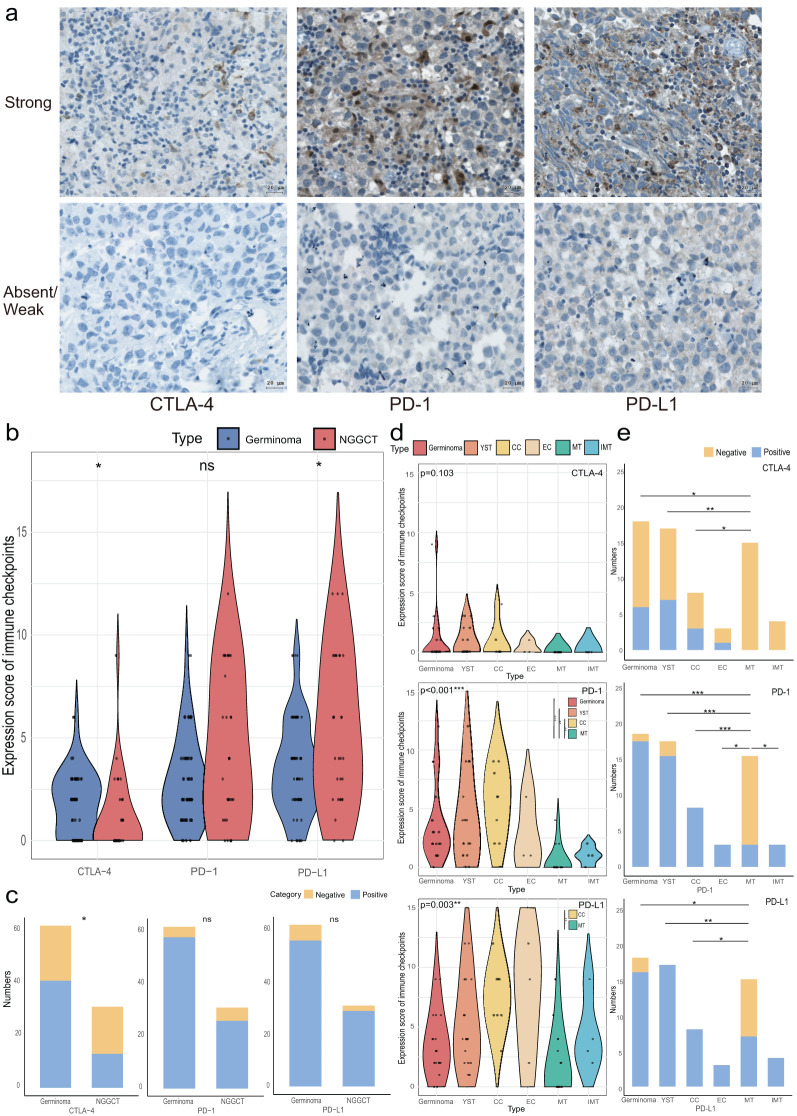
Immune checkpoint expression in CNS GCTs. **(A)** Representative immunohistochemical images of immune checkpoint (CTLA-4, PD-1, PD-L1) staining in CNS GCT specimens. Nuclei counterstained with hematoxylin (blue). Original magnification, x40. Scale bar: 20 μm. **(B)** Violin plots of immune checkpoint expression scores in Germinomas and NGGCTs. **(C)** Stacked bar chart of immune checkpoint expression characteristics in Germinomas and NGGCTs. **(D)** Violin plots of immune checkpoint expression scores across histological subtype regions in NGGCTs. **(E)** Stacked bar chart of immune checkpoint expression characteristics across histological subtype regions in NGGCTs. *p<0.05, **p<0.01, ***p<0.001. ns, non-significant; YST, yolk sac tumor; CC, choriocarcinoma; EC, embryonal carcinoma; MT, mature teratoma; IMT, immature teratoma.

Secondly, we quantified the expression levels of CTLA-4, PD-1, and PD-L1 in each tumor sample, with representative IHC staining images shown in **Figure 2A**. CTLA-4 was positively expressed in 58.06% (54/93) of the samples, while PD-1 and PD-L1 were positive in 90.32% (84/93) and 91.40% (85/93), respectively. As illustrated in **Figures 2B, C**, CTLA-4 had a significantly higher positive expression rate and expression level in germinomas compared to NGGCTs (66.13% vs. 41.94%, p=0.026, and median 2 vs. 0, p=0.021). In contrast, PD-L1 expression was significantly higher in NGGCTs (median score 4 vs. 3, p=0.019), and there was a trend toward higher PD-1 expression in NGGCTs (p=0.118). Positive expression rates of PD-1 and PD-L1 did not significantly differ between germinomas and NGGCTs (93.55% vs. 83.87%, p=0.137, and 90.32% vs. 93.55%, p=0.601, respectively).

In NGGCTs, immune checkpoint expression varied across histological subtypes. CTLA-4 expression was absent in both mature and immature teratomas, and there was no significant difference in CTLA-4 levels among germinoma, yolk sac tumor (YST), choriocarcinoma (CC), embryonal carcinoma (EC), mature teratoma (MT), and immature teratoma (IMT). However, the positive expression rates of CTLA-4 in germinoma, YST, and CC (33.33%, 41.18%, and 37.50%, respectively) were significantly higher than in MT. For PD-1, the expression levels in germinoma, YST, and CC were also significantly higher than in MT. The positive expression rates of PD-1 in germinoma, YST, CC, EC, and IMT (94.44%, 88.24%, 100%, 100%, and 100%) were significantly higher than in MT (20.0%). Among all histological subtypes, CC showed a higher PD-L1 expression score than MT, and PD-L1 expression was more frequent in germinoma, YST, and CC compared to MT (88.89%, 100%, and 100% vs. 46.67%). PD-L1 expression was also present in all EC and IMT samples (**Figures 2D, E**).

### 3.3 Relationship between T cell infiltration abundance and immune checkpoint levels

**Figure 3:**
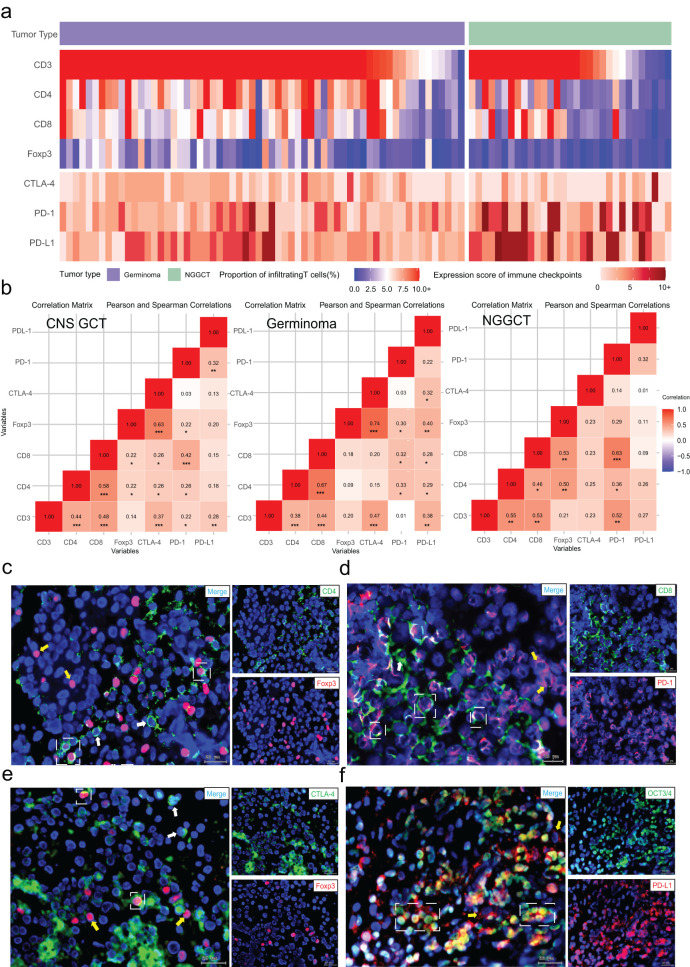
Relationship between T cell subset infiltration and immune checkpoint expression. **(A)** Distribution of T cell subset infiltration and immune checkpoint expression scores in CNS GCTs. **(B)** Correlation matrices of T cell infiltration and immune checkpoint expression in CNS GCTs, Germinomas, and NGGCTs. **(C)** Fluorescent co-staining of CD4 (green) and Foxp3 (red) in tumor regions. White dashed box: CD4+FOXP3+ cells; white arrows: CD4+ cells; yellow arrows: Foxp3+ cells. **(D)** Fluorescent co-staining of CD8 (green) and PD-1 (red) in tumor regions. White dashed box: CD8+PD-1+ cells; white arrows: CD8+ cells; yellow arrows: PD-1+ cells. **(E)** Fluorescent co-staining of CTLA-4 (green) and Foxp3 (red) in tumor regions. White dashed box: Foxp3+CTLA-4+ cells; white arrows: CTLA-4+ cells; yellow arrows: Foxp3+ cells. **(F)** Fluorescent co-staining of OCT3/4 (green) and PD-L1 (red) in tumor regions. White dashed box: OCT3/4+PD-L1+ cells; yellow arrows: PD-L1+ cells. Nuclei stained with DAPI (blue). Original magnification, x40. Scale bar: 20 μm. *p<0.05, **p<0.01, ***p<0.001.

We next examined the correlation between T cell infiltration and immune checkpoint expression in CNS GCTs, with the heat map shown in **Figure 3A**. A small subset of both germinomas and NGGCTs (8.06%, 5/62, and 9.68%, 3/31, respectively) exhibited minimal T cell infiltration and low expression of immune checkpoints (CTLA-4, PD-1, and PD-L1); these NGGCTs were primarily teratomas or tumors with predominant teratoma components. In the overall CNS GCT cohort, CD3+ cell infiltration positively correlated with CD4+ and CD8+ cells (p<0.001), and CD4+ cell abundance correlated with CD8+ and Foxp3+ cells (p<0.001 and p=0.034). All T cell subsets were significantly correlated with CTLA-4 and PD-1 expression (p<0.05), and CD3+ cells also correlated with PD-L1 expression (p=0.007). In germinomas, CD3+ and Foxp3+ cells strongly correlated with CTLA-4 expression (p<0.001), and CD4+, CD8+, and Foxp3+ cells with PD-1 expression (p<0.05). All T cell subsets were positively correlated with PD-L1 expression (p<0.05). In NGGCTs, similar correlations were found among T cell subsets and immune checkpoints (**Figure 3B**).

Additionally, immunofluorescence co-staining revealed significant infiltration of CD4+Foxp3+ regulatory T cells within the tumor immune microenvironment of CNS GCTs, providing initial evidence of an immunosuppressive state in these tumors. A majority of CD8+ T cells expressed PD-1, suggesting that these cells may be in a state of functional exhaustion. Some Foxp3+ cells also exhibited surface CTLA-4 expression, enhancing Treg inhibition of effector T cells. PD-L1 was highly expressed on tumor cells, potentially allowing immune evasion (**Figures 3C–F**, **Supplementary Figure S2**).

These findings demonstrated the complexity and heterogeneity of the CNS GCT immune microenvironment and highlighted its immunosuppressive features.

### 3.4 Correlation of T cell infiltration and immune checkpoint expression with clinical characteristics

**Figure 4:**
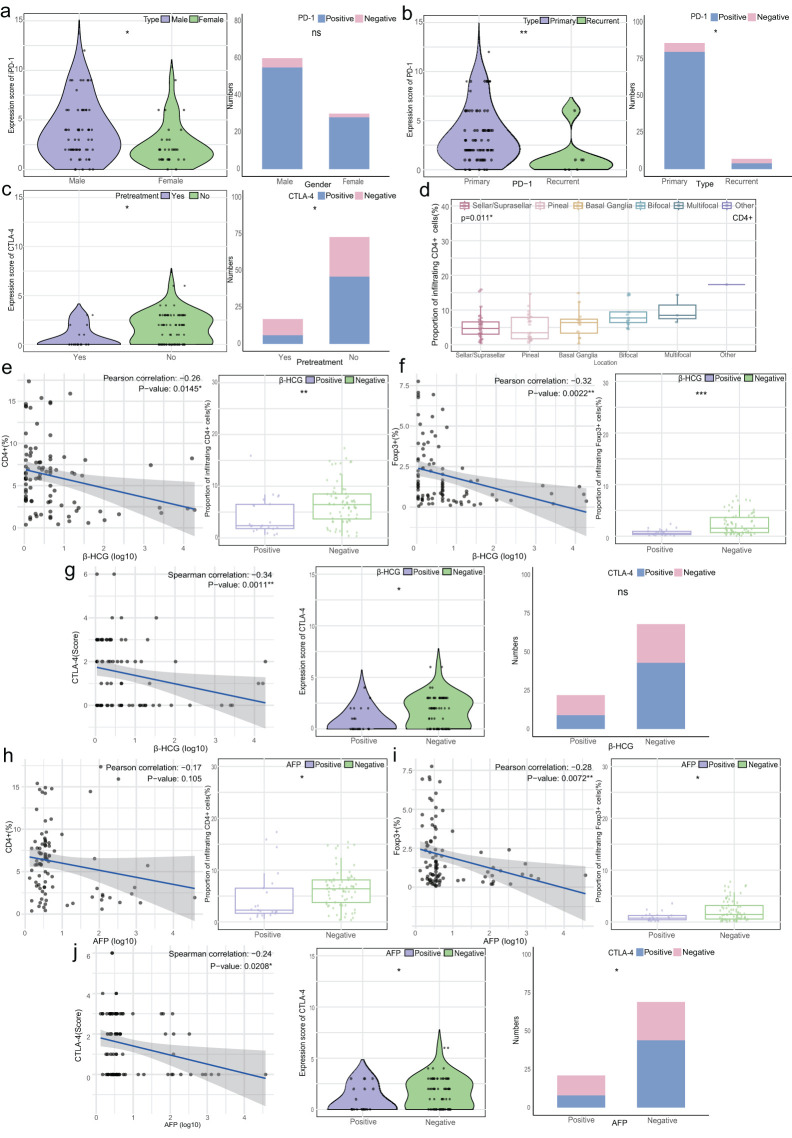
Relationship between T cell infiltration, immune checkpoint expression, and clinical characteristics. **(A)** Left: Violin plot of PD-1 expression scores by gender; Right: Stacked bar chart of PD-1 expression characteristics by gender. **(B)** Left: Violin plot of PD-1 expression scores by disease type (primary/recurrence); Right: Stacked bar chart of PD-1 expression by disease type. **(C)** Left: Violin plot of CTLA-4 expression scores by pretreatment status; Right: Stacked bar chart of CTLA-4 expression by pretreatment status. **(D)** Box plots of CD4+ T cell infiltration in CNS GCTs by location (overall p=0.011, no significant differences between groups). **(E)** Left: Correlation between β-HCG levels (log 10) and CD4+ T cell infiltration (%); Right: Box plot of CD4+ T cell infiltration by β-HCG status. **(F)** Left: Correlation between β-HCG levels (log 10) and Foxp3+ T cell infiltration (%); Right: Box plot of Foxp3+ T cell infiltration by β-HCG status. **(G)** Left: Correlation between β-HCG levels (log 10) and CTLA-4 expression scores; Middle: Violin plot of CTLA-4 expression by β-HCG status; Right: Stacked bar chart of CTLA-4 expression by β-HCG status. **(H)** Left: Correlation between AFP levels (log 10) and CD4+ T cell infiltration (%); Right: Box plot of CD4+ T cell infiltration by AFP status. **(I)** Left: Correlation between AFP levels (log 10) and Foxp3+ T cell infiltration (%); Right: Box plot of Foxp3+ T cell infiltration by AFP status. **(J)** Left: Correlation between AFP levels (log 10) and CTLA-4 expression scores; Middle: Violin plot of CTLA-4 expression by AFP status; Right: Stacked bar chart of CTLA-4 expression by AFP status. *p<0.05, **p<0.01, ***p<0.001. ns, non-significant.

Upon analyzing the relationship between clinical characteristics, T cell subset infiltration, and immune checkpoint expression, it was observed that male patients exhibited higher PD-1 expression than females (median 3 vs. 2, p=0.010), though positive rates were similar (91.67% vs. 93.33%, **Figure 4A**). Additionally, Primary tumors had significantly higher PD-1 levels and positive rates compared to recurrent tumors (median 2.5 vs. 1, p=0.006, and 93.02% vs. 57.14%, p=0.018). Patients without preoperative treatment had higher CTLA-4 expression and positive rates than those with preoperative therapy (median 2 vs. 0, p=0.045, and 63.01% vs. 35.29%, p=0.037). CD4+ cell infiltration differed significantly across tumor sites (p=0.011, **Figures 4B–D**).

CD4+ and Foxp3+ cell abundance showed a weak negative correlation with β-HCG levels (R=-0.26, p=0.0145 and R=-0.32, p=0.0022), with higher infiltration in the β-HCG-negative group (p<0.01, **Figures 4E, F**). CTLA-4 expression was similarly higher in the β-HCG-negative group (p=0.043), though positive rates did not differ significantly (**Figure 4G**). A weak negative correlation was found between AFP levels and Foxp3+ cell abundance (R=-0.28, p=0.0072), with higher infiltration in AFP-negative patients (p<0.05). CTLA-4 expression was also higher in AFP-negative patients (p=0.049, **Figures 4H–J**).

In summary, T cell infiltration and immune checkpoint expression were closely linked to gender, tumor status, treatment history, and tumor marker levels, underscoring their clinical relevance in CNS GCTs.

### 3.5 Association of T cell infiltration and immune checkpoint expression with prognosis

**Figure 5:**
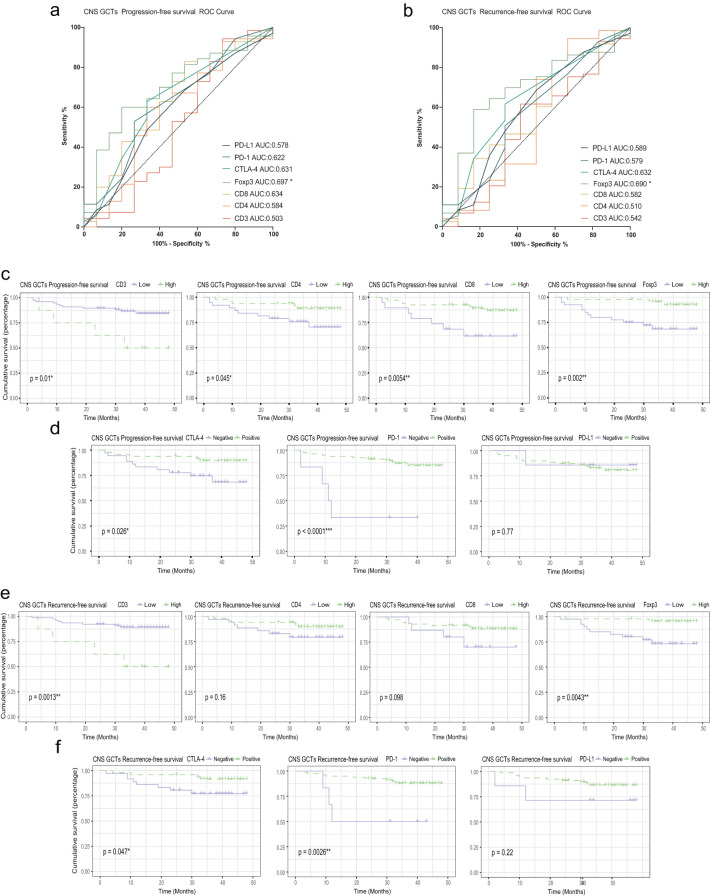
Relationship between PFS, RFS, T cell infiltration, and immune checkpoint expression. **(A)** ROC curve for PFS in CNS GCT patients. **(B)** ROC curve for RFS in CNS GCT patients. **(C)** KM curves for PFS by high vs. low infiltration of T cell subsets. **(D)** KM curves for PFS by immune checkpoint expression. **(E)** KM curves for RFS by high vs. low infiltration of T cell subsets. **(F)** KM curves for RFS by immune checkpoint expression. PFS: Progression-Free Survival; RFS: Relapse-Free Survival; KM curve: Kaplan-Meier survival curve. *p<0.05. **p<0.01. ***p<0.001. ns, non-significant.

During follow-up, we collected prognostic data on overall survival (OS), progression-free survival (PFS), and recurrence-free survival (RFS) for the cohort. ROC analysis showed that Foxp3+ cell infiltration was significantly associated with PFS (p=0.017, AUC=0.697) and RFS (p=0.036, AUC=0.690), while CD3+, CD4+, and CD8+ cell infiltration showed no significant impact on these outcomes. Foxp3+ cell infiltration was also significantly correlated with OS (p=0.0095, AUC=0.886). Patients with high CD3+ infiltration had worse PFS and RFS (p=0.01 and p=0.0013), whereas low CD4+, CD8+, and Foxp3+ infiltration was associated with worse PFS (p=0.045, p=0.0054, and p=0.002) and OS (p=0.048 and p=0.0073). CTLA-4 and PD-1 expression were linked to better PFS and RFS (p<0.05), but PD-L1 did not significantly affect outcomes. (**Figure 5**; **Supplementary Figure S3**).

Univariate analysis revealed that diagnosis, preoperative treatment, tumor location, CD3+, CD8+, and Foxp3+ cell infiltration, as well as CTLA-4 and PD-1 expression, significantly impacted PFS. In multivariate analysis, PD-1 expression emerged as an independent prognostic factor for PFS (HR: 0.127, 95%CI: 0.022-0.716, and p=0.019) and RFS (HR: 0.114, 95%CI: 0.021-0.608, and p=0.011). OS was influenced by tumor location and AFP status in univariate analysis, though these were not independent predictors in multivariate analysis. (**Tables 1**, **2** and **Supplementary Table S4**).

**Table 1:** Univariate and multivariate cox regression analyses for progression-free survival of CNS GCTs patients.

Characteristics	Univariate		Multivariate	
	HR (95%CI)	P	HR (95%CI)	P
Gender				
Male	Reference			
Female	0.745 (0.237-2.342)	0.614		
Age group				
<18	Reference			
≥18	1.421 (0.485-4.159)	0.522		
Diagnosis				
Germinoma	Reference		Reference	
NGGCT	4.733 (1.652-13.562)	0.004**	1.977 (0.432-9.053)	0.380
Pre-treatment				
Yes	4.036 (1.403-11.613)	0.01*	1.827 (0.523-6.385)	0.345
No	Reference		Reference	
Tumor location				
Sellar/suprasellar	Reference		Reference	
Pineal	1.778 (0.474-6.668)	0.394	0.473 (0.072-3.093)	0.435
Basal ganglia	1.173 (0.214-6.413)	0.854	1.018 (0.158-6.577)	0.985
Bifocal	0.601 (0.067-5.384)	0.649	0.999 (0.088-11.345)	0.999
Multifocal	6.018 (1.099-32.946)	0.039*	4.380 (0.461-41.583)	0.198
Other	24.911 (2.453-252.956)	0.007**	7.460 (0.277-200.832)	0.232
Beta-HCG				
Positive	0.243 (0.032-1.847)	0.172		
Negative	Reference			
AFP				
Positive	2.589 (0.909-7.374)	0.075		
Negative	Reference			
Intracranial seeding				
Yes	0.901 (0.203-3.993)	0.891		
No	Reference			
Spinal seeding				
Yes	1.734 (0.391-7.691)	0.469		
No	Reference			
CNS seeding				
Yes	0.746 (0.168-3.306)	0.699		
No	Reference			
CD3				
High	4.015 (1.277-12.626)	0.017*	2.731 (0.393-18.955)	0.310
Low	Reference		Reference	
CD4				
High	0.350 (0.119-1.025)	0.056		
Low	Reference			
CD8				
High	0.259 (0.093-0.721)	0.010*	0.495 (0.108-2.266)	0.365
Low	Reference		Reference	
Foxp3				
High	0.169 (0.047-0.605)	0.006**	0.435 (0.090-2.094)	0.299
Low	Reference		Reference	
CTLA-4				
Positive	0.313 (0.106-0.918)	0.034*	0.688 (0.118-4.000)	0.677
Negative	Reference		Reference	
PD-1				
Positive	0.120 (0.037-0.386)	<0.001***	0.127 (0.022-0.716)	0.019*
Negative	Reference		Reference	
PD-L1				
Positive	1.346 (0.177-10.242)	0.774		
Negative	Reference			

HCG, human chorionic gonadotophin; AFP, alpha-fetoprotein; CNS, central nervous system; CNS GCTs—central nervous system germ cell tumors.

*p<0.05; **p<0.01; ***p<0.001.

**Table 2:** Univariate and multivariate cox regression analyses recurrence-free survival of CNS GCTs patients.

Characteristics	Univariate		Multivariate	
	HR (95%CI)	P	HR (95%CI)	P
Gender				
Male	Reference			
Female	1.062 (0.319-3.534)	0.922		
Age group				
<18	Reference			
≥18	1.374 (0.414-4.565)	0.604		
Diagnosis				
Germinoma	Reference		Reference	
NGGCT	4.035 (1.257-12.947)	0.019*	4.360 (0.833-22.804)	0.081
Pre-treatment				
Yes	3.880 (1.216-12.381)	0.022*	1.654 (0.435-6.290)	0.461
No	Reference		Reference	
Tumor location				
Sellar/suprasellar	Reference		Reference	
Pineal	1.366 (0.273-6.821)	0.704	0.412 (0.055-3.056)	0.385
Basal ganglia	0.837 (0.087-8.049)	0.878	0.920 (0.080-10.623)	0.947
Bifocal	1.829 (0.305-10.955)	0.508	5.660 (0.601-53.293)	0.13
Multifocal	8.359 (1.393-50.179)	0.020*	4.758 (0.464-48.806)	0.189
Other	47.147 (3.920-567.107)	0.002**	6.760 (0.378-120.919)	0.194
Beta-HCG				
Positive	0.305 (0.039-2.364)	0.256		
Negative	Reference			
AFP				
Positive	2.635 (0.828-8.387)	0.101		
Negative	Reference			
Intracranial seeding				
Yes	1.210 (0.265-5.521)	0.806		
No	Reference			
Spinal seeding				
Yes	2.348 (0.514-10.723)	0.271		
No	Reference			
CNS seeding				
Yes	0.998 (0.219-4.557)	0.998		
No	Reference			
CD3				
High	5.713 (1.718-18.993)	0.004**	3.377 (0.528-21.594)	0.199
Low	Reference		Reference	
CD4				
High	0.447 (0.141-1.412)	0.170		
Low	Reference			
CD8				
High	0.376 (0.112-1.259)	0.113		
Low	Reference			
Foxp3				
High	0.146 (0.032-0.672)	0.013*	0.170 (0.025-1.144)	0.069
Low	Reference		Reference	
CTLA-4				
Positive	0.314 (0.094-1.048)	0.060		
Negative	Reference			
PD-1				
Positive	0.167 (0.045-0.623)	0.008**	0.114 (0.021-0.608)	0.011*
Negative	Reference		Reference	
PD-L1				
Positive	0.399 (0.087-1.824)	0.236		
Negative	Reference			

Abbreviations: HCG--human chorionic gonadotophin; AFP--alpha-fetoprotein; CNS:central nervous system; CNS GCTs—central nervous system germ cell tumors.

* :p<0.05; ** :p<0.01.

## 4 Discussion

In recent years, significant progress has been made in understanding the pathogenic mechanisms of CNS GCTs, particularly in genetics, including chromosomal variations and genomic alterations (21–27). As a unique and complex tumor type, increasing attention has been directed toward the tumor microenvironment (TME) of CNS GCTs. A deeper understanding of the TME is essential for advancing therapeutic approaches.

In this study, we observed significant T cell infiltration in the majority of both germinoma and NGGCT cases, with notable heterogeneity in the extent of T cell infiltration across individual cases. Several studies have confirmed that a prominent feature of the germinoma tumor microenvironment is the infiltration of large numbers of immune cells. Saito et al. reported that 70-80% of infiltrating lymphocytes in germinomas are T cells (16). Another study found that T cells play a major role in the tumor microenvironment, accounting for about 30.9% of all lymphocytes, with considerable variation in immune cell infiltration across cases (13). Additionally, CD3+ T cells were found to dominate the immune microenvironment of germinomas, with helper T cells (Th cells) outnumbering cytotoxic T cells (Tc cells) (17). Similarly, abundant immune cell infiltration, particularly a high proportion of T cells, has been observed in germ cell tumors of other systems, such as testicular seminomas (7, 10, 11). This extensive immune cell infiltration is considered one of the “two cell patterns” in the histopathology of germ cell tumors. Regarding immune checkpoints, our study revealed that CTLA-4 was positively expressed in over 60% of germinoma cases and more than 40% of NGGCT cases. Li B et al. also reported significant CTLA-4 expression in the immune-hot subtype of CNS GCTs, 94.4% of which were germinomas (27). A study on testicular germ cell tumors (TGCTs) also showed high CTLA-4 expression in both seminoma and non-seminoma cases (seminoma: 87.2%; non-seminoma: 79.5%) (10), a higher proportion than observed in our cohort. Furthermore, we found that PD-1 and PD-L1 were highly expressed in both germinoma and NGGCT cases, consistent with previous studies on intracranial and extracranial germ cell tumors (10, 13–15).

Amid the significant T cell infiltration and immune checkpoint expression observed, CNS GCTs exhibit clear features of an immunosuppressive tumor microenvironment. First, regulatory T cells (Tregs) typically suppress immune responses by inhibiting the activity of functional T cells within the tumor microenvironment (28, 29). In CNS GCTs, we observed a substantial presence of CD4+Foxp3+ Treg cells, with some Foxp3+ cells also expressing CTLA-4, suggesting that these Tregs actively contribute to the immunosuppressive microenvironment. The expression of CTLA-4 may further amplify this effect, particularly in germinomas. Second, the infiltration levels of different T cell subsets were positively correlated with the expression of immune checkpoints (CTLA-4, PD-1). Immunofluorescence co-staining revealed that a significant number of CD8+ T cells expressed PD-1 on their surface, while PD-L1 expression was closely associated with malignant subtypes (except teratomas). These findings indicate that the high activity of the PD-1/PD-L1 axis allows tumor cells to evade anti-tumor immune responses, likely playing a more prominent role in NGGCTs. Moreover, this heightened activity may lead to effector T cell exhaustion, ultimately resulting in an immune-tolerant microenvironment. Lastly, in germinomas, the proportion of CD4+ Th cells exceeded that of CD8+ Tc cells, possibly indicating that cytotoxic T cells are not fully functional, leading to poor tumor control. However, this pattern was not observed in NGGCTs, likely due to the high heterogeneity of these tumors, which are often mixed and comprised of multiple histological subtypes. Given the complexity of their immune microenvironment, further investigation into the immunological dynamics of NGGCTs is warranted.

The tumor microenvironment, characterized by high T cell infiltration and significant immune checkpoint expression in CNS GCTs, is not only closely linked to the tumor’s biological behavior but may also directly impact patient outcomes and prognosis. In this study, we observed differences in PD-1 expression levels between genders, although the positive expression rate did not show a significant difference. This suggests that gender may play a distinct role in shaping the immune microenvironment of CNS GCTs. Previous studies have highlighted several gender-related factors influencing the development and progression of CNS GCTs, including chromosomal alterations (23, 25), gender-specific differences in mutation rates within the MAPK pathway (21), and mutations in genes associated with sex hormone receptors (26). Additionally, the potential role of sex hormones in modulating the immune system may contribute to these differences. Future research involving larger cohorts could help determine whether gender-specific disparities exist within the tumor immune microenvironment. Such studies would also further elucidate the impact of gender on tumor immunity and the response to immune checkpoint inhibitors, which is critical for developing personalized treatment strategies. We also observed differences in PD-1 expression between primary and recurrent tumors, as well as the impact of preoperative radiotherapy and chemotherapy on CTLA-4 expression, suggesting that these factors may influence the immune microenvironment. PD-1 may play a more prominent role in primary tumors. As a crucial regulator in the immune response process (30), its upregulation in primary tumors could serve as an indicator of immune activity. However, in recurrent cases, its role may be reduced or supplanted by other mechanisms. This implies that immunosuppressive treatments targeting PD-1 may be more effective in primary tumors, while recurrent cases may require alternative therapeutic strategies to address more complex immunosuppressive and immune evasion mechanisms. Moreover, preoperative treatments may alter the immune status of the tumor microenvironment, potentially affecting the response to immunotherapy. Future studies should explore the impact of these factors on immunotherapy, investigating the differences and similarities in treatment strategies for primary versus recurrent diseases, as well as determining the optimal timing for treatment.

Notably, our study revealed a significant correlation between tumor markers (β-HCG and AFP) and the proportion of infiltrating CD4+ and Foxp3+ cells, as well as CTLA-4 expression. Specifically, the negative correlation between β-HCG and AFP levels and the infiltration of CD4+ and Foxp3+ cells, as well as CTLA-4 expression, suggests that tumors with lower levels of these markers may be associated with a more pronounced immunosuppressive microenvironment. We hypothesize that lower marker levels could be linked to enhanced immune evasion mechanisms, potentially reflected in increased Treg (regulatory T cell) infiltration and elevated CTLA-4 expression, thereby fostering a stronger immunosuppressive environment. This type of microenvironment aids tumor cells in evading host immune surveillance. In contrast, tumors with higher marker levels may employ alternative mechanisms to evade immune responses, which could involve components of the humoral or innate immune systems (9, 13, 17), or be linked to specific molecular features of the tumor, although these mechanisms remain to be fully elucidated. During pregnancy, HCG exhibits a range of immunoregulatory properties, not only modulating the immune microenvironment within the reproductive system but also affecting the function of peripheral immune cells (31, 32). As for AFP, studies have shown that it can significantly influence the immune microenvironment in liver cancer through multiple mechanisms, including its effects on liver cancer stem cells, various immune cells, fibroblasts, and the endothelial-to-mesenchymal transition process (33). However, in germ cell tumors, only a limited number of prospective cohort studies have confirmed the role of β-HCG and AFP in monitoring treatment response and predicting prognosis (34, 35). Their influence on shaping the immune microenvironment remains largely underexplored. Our findings underscore the heterogeneity and complexity of the tumor microenvironment in germ cell tumors. Therefore, understanding the potential impact of tumor markers on the immune microenvironment could offer valuable insights for developing novel immunotherapies. Future research into how tumor markers influence the immune microenvironment in germ cell tumors is both essential and urgently needed.

Previous studies have shown that different levels of lymphocyte infiltration, their subtypes, and immune checkpoint expression can impact patient prognosis in both intracranial and extracranial germ cell tumors. For instance, high lymphocyte infiltration in germinomas has been associated with better outcomes (13), and in TGCTs, high T cell infiltration, low Treg infiltration, and an active PD-1/PD-L1 axis have been linked to favorable prognosis (7, 8, 10, 12). However, in our study, patients with high CD3+ infiltration had worse PFS and RFS, suggesting that increased non-specific T cell infiltration may not correlate with anti-tumor effects. This could be due to an immunosuppressive tumor microenvironment that impairs T cell function. Conversely, patients with low Foxp3+ Treg infiltration had worse OS, PFS and RFS, indicating that Foxp3+ cells may play a critical role in anti-tumor immunity and are important for controlling CNS GCT progression (28, 36). These findings warrant further investigation, particularly regarding the specific roles of CD3+ and Foxp3+ cells in tumor progression and their interactions with tumor cells and other immune cells.CD4+ and CD8+ T cells are generally regarded as the central players in anti-tumor immunity (37, 38). In this study, we observed that low CD4+ and CD8+ cell infiltration was associated with poorer PFS, with low CD8+ infiltration particularly linked to worse OS. This further suggests that CD4+ and CD8+ T cells contribute to tumor immune defense. Although the infiltration characteristics of various T cell subtypes were correlated with patient prognosis to varying degrees, none demonstrated significant independent prognostic value. This suggests that specific immunosuppressive mechanisms, such as Treg cell infiltration and high immune checkpoint expression, may be present in CNS GCTs, preventing T cells from exerting their full anti-tumor effects. The presence and underlying mechanisms of these suppressive factors warrant further investigation. Patients with negative CTLA-4 expression showed worse PFS and RFS, although CTLA-4 was not identified as an independent prognostic factor. Similarly, PD-L1 expression did not significantly correlate with patient prognosis, a finding that requires further validation.

However, a key discovery in our study is that PD-1 expression was an independent prognostic factor for PFS and RFS in CNS GCT patients. This not only underscores the potential of PD-1 in risk stratification and subgroup identification of patients, but also emphasizes the promise of anti-PD-1 immunotherapy for CNS GCT, especially in patients with high T-cell infiltration, positive PD-1 expression, and resistance to other treatments. At the same time, considering the current research foundation and treatment status, it is important to interpret our results with caution. Although we observed T cell infiltration and a correlation between immune checkpoints, particularly PD-1, and the clinical features and prognosis of CNS GCT patients, it remains unclear whether positive or elevated expression of immune checkpoints signifies the suitability of immune checkpoint inhibitor therapy. So far, the expression of immune checkpoints, including CTLA-4 and PD-L1, has not been able to predict the response of germ cell tumor patients to immune checkpoint inhibitors (10, 39). A similar lack of predictive value has also been observed in renal-cell carcinoma (40, 41). This is due to our incomplete understanding of the intrinsic relationship between immune checkpoint expression and the response to immunotherapy. Several attempts have been made to treat extracentral germ cell tumors with ICB (42–46), with results that have been both promising and discouraging. While some TGCT patients have not experienced benefits from immunotherapy, other studies have yielded encouraging results. Reports have indicated that monotherapy with PD-1 monoclonal antibodies in germinoma patients, as well as combination therapy with PD-1 and CTLA-4 monoclonal antibodies in CNS NGGCT patients, both demonstrated some therapeutic efficacy (27, 47). In a study involving PD-1 antibody treatment for 7 patients with extracranial GCTs who relapsed after high-dose chemotherapy and stem cell transplantation, 3 patients received at least 6 months of PD-1 therapy, and two of them achieved long-term tumor responses (43). Thus, we are optimistic about the potential future clinical application of immunosuppressive therapy in CNS GCT patients. Therefore, whether immune checkpoint inhibitor therapy can genuinely improve the prognosis of CNS GCT patients still requires validation through future clinical trials. Secondly, the differences in immune microenvironment characteristics between responders and non-responders to ICB therapy need to be elucidated. Further research is required to identify the specific features that can reliably predict which patients are more likely to benefit from immune checkpoint inhibitor treatment. The precise assessment of the applicability of immunosuppressive therapy, along with accurate patient identification methods, will provide the solution to this issue. This study employs IHC to assess the expression and levels of immune checkpoints in CNS GCT patients, a method not yet part of routine clinical practice. However, due to its applicability and cost-effectiveness, coupled with the findings of this study, it could be further validated in the future for its potential role in patient risk stratification. In addition, the optimal use of immunosuppressive agents in the future requires further investigation. Given the presence of the blood-brain barrier (BBB), the ability of immune checkpoint inhibitors to penetrate the BBB must be considered. Some studies have explored the use of ICB drugs to treat recurrent high-grade gliomas through intraoperative intracranial injection and postoperative Ommaya reservoir delivery (48), offering a novel approach for further investigation. Should ICB be used as monotherapy or in combination with other immune checkpoint inhibitors or agents (such as cytokines or immunosuppressive steroids), and how might this impact the therapeutic efficacy of ICB? Additionally, how can we mitigate immune-related adverse events (irAEs) associated with ICB therapy (49)? These are important questions that require further investigation in future research. Moreover, for patients who cannot undergo immunosuppressive therapy or who experience treatment failure, Car-T therapy—demonstrating some efficacy in TGCT treatment (39)—may offer an alternative immunotherapy option.

In addition to the inherent limitations of retrospective studies, another significant limitation of this study is the reliance on FFPE specimens due to the lack of sufficient fresh tissue samples, given current diagnostic and treatment practices. This constrained our ability to conduct detailed analyses of the specific types, functional changes, and spatiotemporal evolution of infiltrating T cells in the CNS GCT tumor microenvironment, as well as the dynamic changes in immune checkpoint expression. Furthermore, FFPE samples may lead to antigen modification, degradation, or denaturation, which could affect the IHC staining results. Additionally, methods like IHC and IF are inherently prone to subjective interpretation. To address these issues, we employed a robust antigen retrieval method, including appropriate control groups, and utilized as objective a quantification approach as possible to minimize potential biases. Moreover, the follow-up period for the cases included in this cohort was relatively short, which may limit the ability to make long-term prognostic interpretations, particularly regarding PFS and RFS. The majority of NGGCT cases in this study were mixed germ cell tumors, which may not fully represent the diversity of single histological subtypes found in NGGCTs. This limitation reduces the generalizability of the findings to all NGGCT subtypes. Since mixed germ cell tumors constitute a large proportion of NGGCT cases, future studies with larger cohorts and longer follow-up periods are needed to better assess the prognostic significance of immune infiltration and checkpoint expression across different histological subtypes and to evaluate their long-term prognostic value. Due to the scarcity of CNS GCT samples, future collaborations across multiple centers and regions will be essential for further advancing research in this area.

## 5 Conclusion

In conclusion, our study highlighted the characteristics of T cell infiltration and significant immune checkpoint expression in CNS GCTs, revealing the highly heterogeneous and immunosuppressive nature of the tumor microenvironment. We demonstrated that T cell infiltration and immune checkpoint expression are closely associated with patients’ clinical characteristics and prognosis. Notably, PD-1 expression was identified as an independent prognostic predictor, providing a foundation for improving risk stratification in CNS GCT patients and supporting the potential for future clinical applications of immune checkpoint inhibitors, such as PD-1 monoclonal antibodies.

## Data Availability

The original contributions presented in the study are included in the article/**Supplementary Material**, further inquiries can be directed to the corresponding author/s.
