# Supplementary material for: The clinical significance of T cell infiltration and immune checkpoint expression in central nervous system germ cell tumors
Source: Front Immunol. 2025 Jan 31;16:1536722. doi: 10.3389/fimmu.2025.1536722 (PMC11825448; doi:10.3389/fimmu.2025.1536722)
Supplement: Supplementary file 1 [file DataSheet1.pdf]

Figure S1. Clinical characteristics of cohort.

a. Distribution of patients of gender and age. b. Box plots of patient age in Germinomas and NGGCTs. c. Pie chart of the distribution of tumor locations. d. Stacked bar chart of the distribution of tumor locations in Germinoma and NGGCT patients. e. f. g. KM curves of OS, PFS, and RFS in Germinoma and NGGCT patients. OS: Overall Survival; PFS: Progression-Free Survival; RFS: Relapse-Free Survival; KM curve: Kaplan–Meier survival curve.

Figure S2. Fluorescent co-staining of malignant histological component markers and PD-L1 in NGGCTs.

a. Fluorescent co-staining of AFP (green) and PD-L1 (red) in tumor regions. b. Fluorescent co-staining of HCG (green) and PD-L1 (red) in tumor regions. c. Fluorescent co-staining of CD30 (green) and PD-L1 (red) in tumor regions. Nuclei stained with DAPI (blue). Original magnification, x40. Scale bar: 20 µm.

Figure S3. Relationship between OS, T cell infiltration, and immune checkpoint expression.

a. ROC curve for OS in CNS GCT patients. b. KM curves for OS by high vs. low infiltration of T cell subsets. d. KM curves for OS by immune checkpoint expression. OS: Overall survival; KM curve: Kaplan–Meier survival curve. *p<0.05. **p<0.01. ***p<0.001.

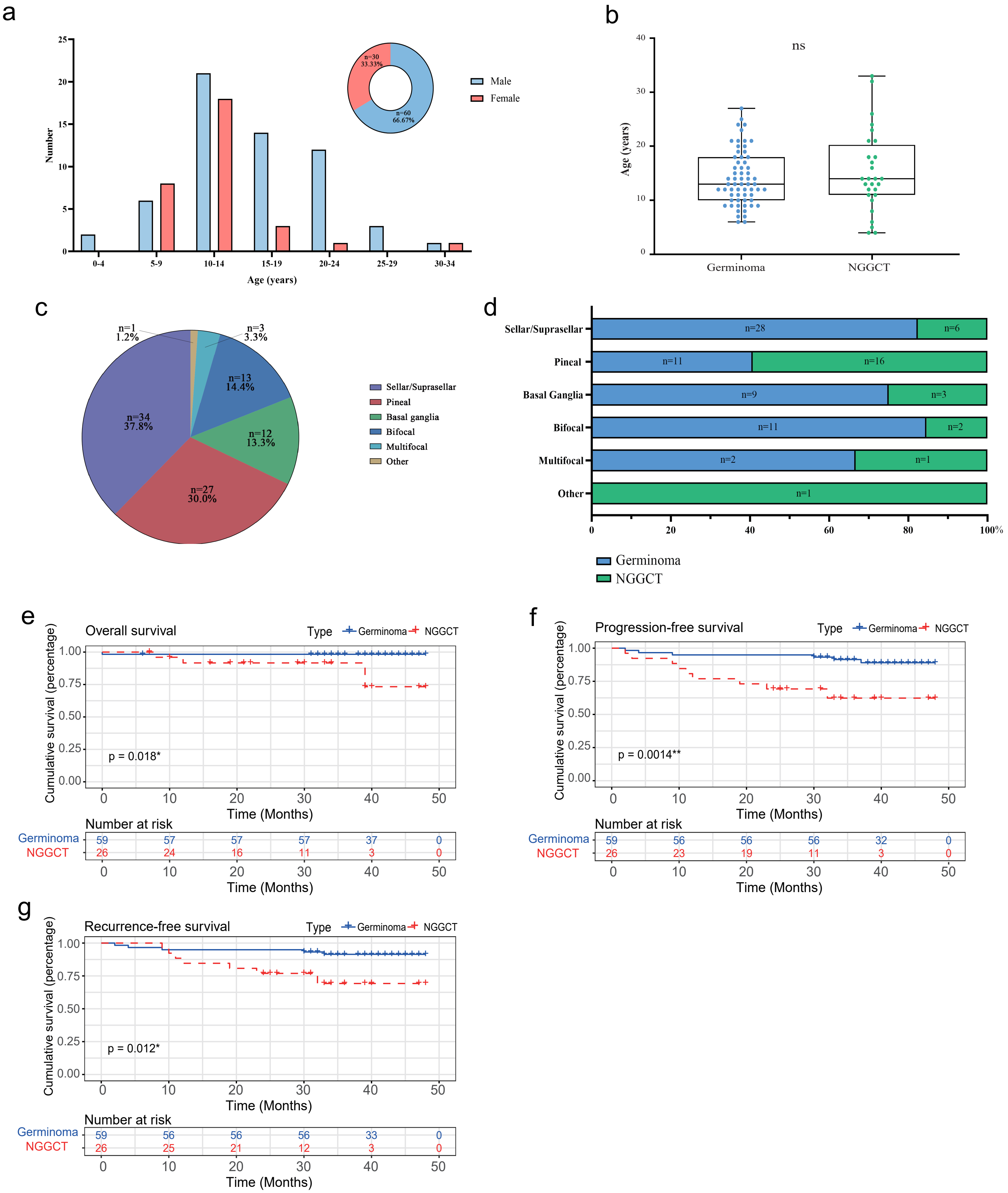
a
Number
Age (years)
0-4
5-9
10-14
15-19
20-24
25-29
30-34
n=30
33.33%
n=60
66.67%
Male
Female
b
ns
Age (years)
Germinoma
NGGCT
c
n=1
1.2%
n=3
3.3%
n=13
14.4%
n=34
37.8%
n=12
13.3%
n=27
30.0%
Sellar/Suprasellar
Pineal
Basal ganglia
Bifocal
Multifocal
Other
d
Sellar/Suprasellar
n=28
n=6
Pineal
n=11
n=16
Basal Ganglia
n=9
n=3
Bifocal
n=11
n=2
Multifocal
n=2
n=1
Other
n=1
0
20
40
60
80
100%
Germinoma
NGGCT
e
Overall survival
Type
Germinoma
NGGCT
Cumulative survival (percentage)
p = 0.018*
Time (Months)
Number at risk
Germinoma 59 57 57 57 37 0
NGGCT 26 24 16 11 3 0
Time (Months)
f
Progression-free survival
Type
Germinoma
NGGCT
Cumulative survival (percentage)
p = 0.0014**
Time (Months)
Number at risk
Germinoma 59 56 56 56 32 0
NGGCT 26 23 19 11 3 0
Time (Months)
g
Recurrence-free survival
Type
Germinoma
NGGCT
Cumulative survival (percentage)
p = 0.012*
Time (Months)
Number at risk
Germinoma 59 56 56 56 33 0
NGGCT 26 25 21 12 3 0
Time (Months)

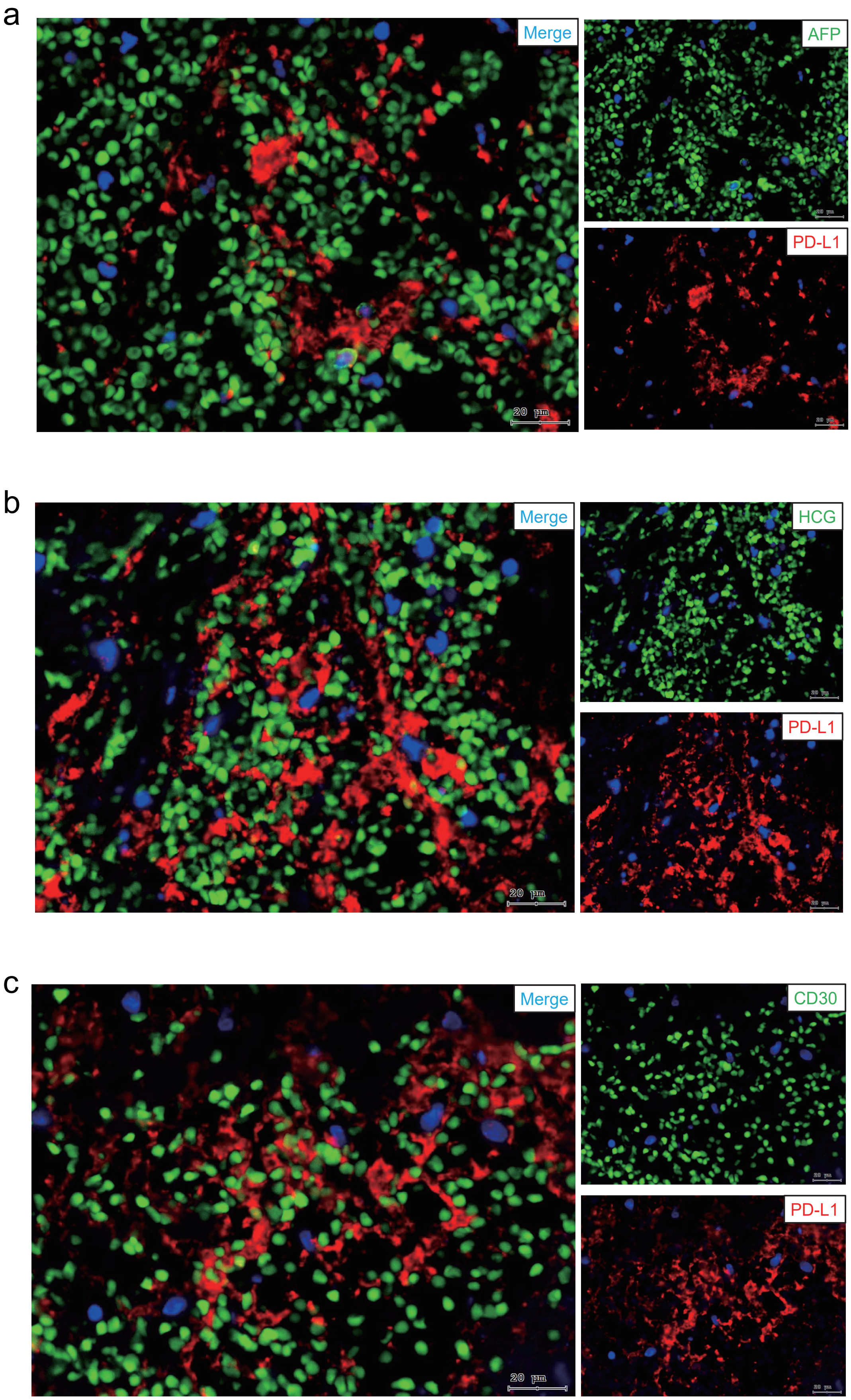
a
Merge
AFP
PD-L1
20 μm
b
Merge
HCG
PD-L1
20 μm
c
Merge
CD30
PD-L1
20 μm

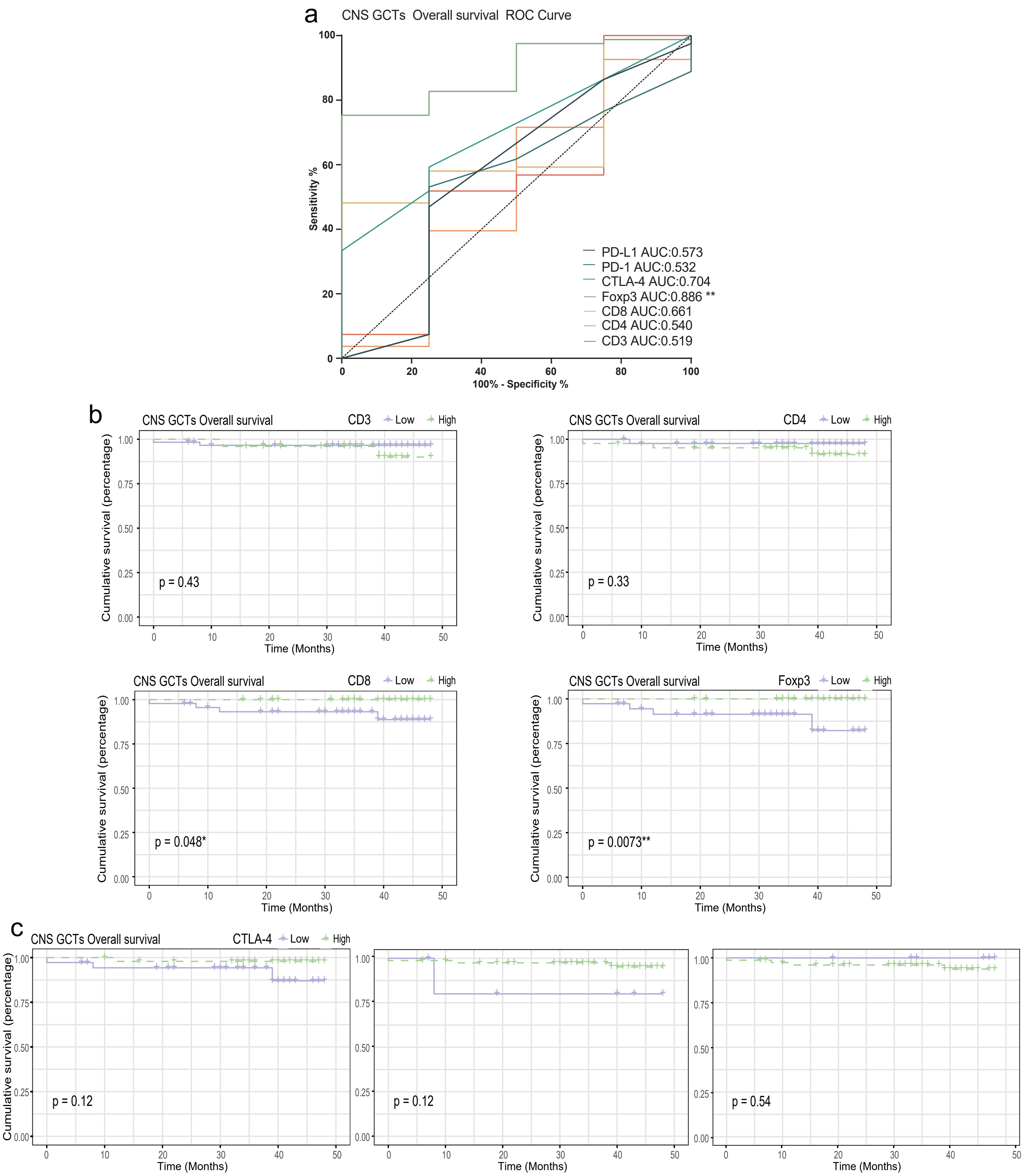

a
CNS GCTs Overall survival ROC Curve
Sensitivity %
100% - Specificity %
PD-L1 AUC:0.573
PD-1 AUC:0.532
CTLA-4 AUC:0.704
Foxp3 AUC:0.886 **
CD8 AUC:0.661
CD4 AUC:0.540
CD3 AUC:0.519
b
CNS GCTs Overall survival
CD3 Low High
p = 0.43
CNS GCTs Overall survival
CD4 Low High
p = 0.33
CNS GCTs Overall survival
CD8 Low High
p = 0.048*
CNS GCTs Overall survival
Foxp3 Low High
p = 0.0073**
Cumulative survival (percentage)
Time (Months)
c
CNS GCTs Overall survival
CTLA-4 Low High
p = 0.12
p = 0.12
p = 0.54
Cumulative survival (percentage)
Time (Months)
