# Supplementary material for: The clinical significance of T cell infiltration and immune checkpoint expression in central nervous system germ cell tumors
Source: Front Immunol. 2025 Jan 31;16:1536722. doi: 10.3389/fimmu.2025.1536722 (PMC11825448; doi:10.3389/fimmu.2025.1536722)
Supplement: Supplementary file 2 [file DataSheet2.pdf]

Supplementary TableS1. Clinical characteristics of the cohort.

**Clinical Characteristics**	**All**		**Germinomas**		**NGGCTs**		***P***
	**n=90**	**%**	**n=62**	**%**	**n=28**	**%**	
Age, years old							
<18	65	72.22	46	74.19	19	67.86	0.534
>=18	25	27.78	16	25.81	9	32.14	
Gender, n %							
Male	60	66.67	36	58.06	24	85.71	0.01*
Female	30	33.33	26	41.94	4	14.29	
Pre-treatment							
Yes	73	81.11	59	95.16	14	50.00	<0.001***
No	17	18.89	3	4.84	14	50.00	
Disease type							
Primary	86	95.56	60	96.77	26	92.86	0.586
Recurrent	4	4.44	2	3.23	2	7.14	
Locations							
Sellar/Suprasellar	34	37.78	29	46.77	5	17.86	0.001**
Pineal	27	30.00	11	17.74	16	57.14	
Basal ganglia	12	13.33	9	14.52	3	10.71	
Bifocal	13	14.44	11	17.74	2	7.14	
Multifocal	3	3.33	2	3.23	1	3.57	
Other	1	1.11	0	0.00	1	3.57	
Beta-HCG							
Positive	22	24.44	9	14.52	13	46.43	0.001**
Negative	68	75.56	53	85.48	15	53.57	
AFP							
Positive	21	23.33	3	4.84	18	64.29	<0.001***
Negative	69	76.67	59	95.16	10	35.71	
Intracranial Seeding							
Yes	12	13.33	10	16.13	2	7.14	0.328
No	78	86.67	52	83.87	26	92.86	
Spinal Seeding							
Yes	7	7.78	5	8.06	2	7.14	>0.999
No	83	92.22	57	91.94	26	92.86	
CNS Seeding							
Yes	14	15.56	11	17.74	3	10.71	0.536
No	76	84.44	51	82.26	25	89.29	

Abbreviations: HCG--human chorionic gonadotophin; AFP--alpha-fetoprotein; CNS--central nervous system.
