# Supplementary material for: The clinical significance of T cell infiltration and immune checkpoint expression in central nervous system germ cell tumors
Source: Front Immunol. 2025 Jan 31;16:1536722. doi: 10.3389/fimmu.2025.1536722 (PMC11825448; doi:10.3389/fimmu.2025.1536722)
Supplement: Supplementary file 3 [file DataSheet3.pdf]

Supplementary TableS2. CNS non-germinomatous germ cell tumors and components.

NGGCTs	Component
NGGCT 1	MT Germinoma
NGGCT 2	MT
NGGCT 3 c	MT Germinoma
NGGCT 4	CC Germinoma YST
NGGCT 5	Germinoma YST EC
NGGCT 6	MT IMT Germinoma
NGGCT 7	MT
NGGCT 8	YST MT
NGGCT 9	YST
NGGCT 10	MT YST
NGGCT 11	YST Germinoma
NGGCT 12	IMT
NGGCT 13	MT CC
NGGCT 14	YST Germinoma
NGGCT 15	MT
NGGCT 16	MT
NGGCT 17	MT Germinoma
NGGCT 18	CC Germinoma
NGGCT 19 b	YST Germinoma
NGGCT 20	CC IMT
NGGCT 21	MT Germinoma CC YST EC
NGGCT 22	CC YST EC MT
NGGCT 23	MT Germinoma CC YST
NGGCT 24	CC Germinoma
NGGCT 25 a	Germinoma YST
NGGCT 26	YST Germinoma
NGGCT 27	YST Germinoma IMT
NGGCT 28	YST Germinoma
NGGCT 29* a	YST
NGGCT 30* b	YST
NGGCT 31* c	MT

*:Same patients' recurrent tissue; a, b, c:Same patient.

Abbreviations: CC--choriocarcinoma; EC--embryonal carcinoma; MT--mature teratoma; IMT--immature teratoma; YST--yolk-sac tumor; CNS--central nervous system; NGGCT--non-germinomatous germ cell tumor.
