# Supplementary material for: The clinical significance of T cell infiltration and immune checkpoint expression in central nervous system germ cell tumors
Source: Front Immunol. 2025 Jan 31;16:1536722. doi: 10.3389/fimmu.2025.1536722 (PMC11825448; doi:10.3389/fimmu.2025.1536722)
Supplement: Supplementary file 4 [file DataSheet4.pdf]

Supplementary TableS3. Antibodies Used in the Investigation.

Antibody	Vendor	Clone	Dilution	Application	Identifier
CD3	proteintech	3F3A1	IHC:1:1500	IHC	Cat#60181-1-Ig
CD4	proteintech	2H4A2	IHC:1:500;IF:1:200	IHC;IF	Cat#67786-1-Ig
CD8	proteintech	1G2B10	IHC:1:1000;IF:1:200	IHC;IF	Cat#66868-1-Ig
Foxp3	Abcam	236A/E7	IHC:1:500;IF:1:500	IHC;IF	Cat#ab20034
CTLA-4	Zenbio	ZN249	IHC:1:200	IHC	Cat#M50008
CTLA-4	proteintech	Polyclonal	IF:1:200	IF	Cat#30648-1-AP
PD-1	proteintech	4H4D1	IHC:1:3000;IF:1:200	IHC;IF	Cat#66220-1-Ig
PD-L1	proteintech	2B11D11	IHC:1:3000;IF:1:500	IHC;IF	Cat#66248-1-Ig
OCT3/4 (POU5F1)	Santa Cruz	C-10	IF:1:500	IF	Cat#sc-5279
Beta-HCG	proteintech	Polyclonal	IF:1:500	IF	Cat#11615-1-AP
AFP	proteintech	Polyclonal	IF:1:500	IF	Cat#14550-1-AP
CD30	proteintech	Polyclonal	IF:1:500	IF	Cat#55132-1-AP
