# Supplementary material for: The clinical significance of T cell infiltration and immune checkpoint expression in central nervous system germ cell tumors
Source: Front Immunol. 2025 Jan 31;16:1536722. doi: 10.3389/fimmu.2025.1536722 (PMC11825448; doi:10.3389/fimmu.2025.1536722)
Supplement: Supplementary file 5 [file DataSheet5.pdf]

Supplementary TableS4. Univariate and multivariate cox regression analyses for overall survival of CNS GCTs pat

	Univariate		Multivariate	
**Characteristics**	**HR (95%CI)**	***P***	**HR (95%CI)**	***P***
Gender				
Male	Reference			
Female	0.633(0.065-6.129)	0.693		
Age group				
<18	Reference			
⩾18	2.835(0.399-20.148)	0.298		
Diagnosis				
Germinoma	Reference			
NGGCT	9.673(0.955-98.001)	0.055		
Pre-treatment				
Yes	1.910(0.195-18.716)	0.578		
No	Reference			
Tumor location				
Sellar/suprasellar	Reference		Reference	
Pineal	1.428(0.144-14.112)	0.761	0.996(0.096-10.29)	0.997
Basal ganglia	2.59E-08(0-Inf)	0.999	2.33E-8(0-Inf)	0.998
Bifocal	2.59E-08(0-Inf)	0.999	2.28E-8(0-Inf)	0.998
Multifocal	9.813(1.020-94.402)	0.048*	7.432(0.759-72.820)	0.085
Other	25.978(2.644-255.199)	0.005**	5.416(0.491-59.710	0.168
Beta-HCG				
Positive	1.504(0.153-14.745)	0.726		
Negative	Reference			
AFP				
Positive	11.968(1.215-117.924)	0.033*	8.768(0.865-88.850)	0.066
Negative	Reference		Reference	
Intracranial seeding				
Yes	1.814(0.187-17.554)	0.607		
No	Reference			
Spinal seeding				
Yes	3.138(0.323-30.469)	0.324		
No	Reference			
CNS seeding				
Yes	1.528(0.158-14.756)	0.714		
No	Reference			
CD3				
High	2.170(0.305-15.436)	0.439		
Low	Reference			
CD4				
High	2.911(0.302-28.032)	0.355		
Low	Reference			
CD8				
High	1.71E-9(0-Inf)	0.999		
Low	Reference			
Foxp3				
High	7.30E-10(0-Inf)	0.999		
Low	Reference			
CTLA-4				
Positive	0.198(0.020-1.934)	0.164		
Negative	Reference			
PD-1				
Positive	0.197(0.020-1.902)	0.160		
Negative	Reference			
PD-L1				
Positive	7.58E+07(0-Inf)	0.999		
Negative	Reference			

Abbreviations: HCG--human chorionic gonadotophin; AFP--alpha-fetoprotein; CNS:central nervous system; CNS GCTs--central nervous system germ cell tumors.

* :p<0.05.
